# Expression of the long non-coding RNA TCL6 is associated with clinical outcome in pediatric B-cell acute lymphoblastic leukemia

**DOI:** 10.1038/s41408-019-0258-9

**Published:** 2019-11-25

**Authors:** Marta Cuadros, Álvaro Andrades, Isabel F. Coira, Carlos Baliñas, María I. Rodríguez, Juan Carlos Álvarez-Pérez, Paola Peinado, Alberto M. Arenas, Daniel J. García, Pilar Jiménez, Mireia Camós, Antonio Jiménez-Velasco, Pedro P. Medina

**Affiliations:** 1 Department of Biochemistry and Molecular Biology III and Immunology, University of Granada, Granada, Spain; 2 GENYO, Centre for Genomics and Oncological Research, Pfizer/University of Granada/Andalusian Regional Government, Granada, Spain; 3 Health Research Institute of Granada (ibs.Granada), Granada, Spain; 4 Department of Biochemistry and Molecular Biology I, University of Granada, Granada, Spain; 5 Department of Clinical Analysis and Immunology, UGC Laboratorio Clínico, University Hospital Virgen de las Nieves, Granada, Spain; 6 Hematology Laboratory, Hospital Sant Joan de Déu Barcelona, University of Barcelona; Institut de Recerca Hospital Sant Joan de Deu Barcelona; Centro de Investigación Biomédica en Red de Enfermedades Raras (CIBERER), Instituto de Salud Carlos III, Madrid, Spain; 7 Hematology Laboratory, Universitary Regional Hospital, Málaga, Spain

**Keywords:** Cancer genomics, Acute lymphocytic leukaemia

## Dear Editor,

Acute lymphoblastic leukemia (ALL) is a clinically and biologically heterogeneous disease recurrently affected by chromosomal aberrations, including translocations, amplifications, and aneuploidies^1^. These aberrations have important implications for the diagnosis, sub-classification, prognosis and, overall, for making appropriate therapeutic decisions. The reciprocal translocation t(12;21)(p13;q22)[ETV6/RUNX1] is the most frequent chromosomal rearrangement in pediatric B-cell acute lymphoblastic leukemia (B-ALL) with an incidence of ~25%^2^. This rearrangement, as well as high hyperdiploidy, is associated with a favorable outcome under current treatment protocols, but up to 20% of ETV6-RUNX1-positive pediatric B-ALL patients experience a late disease relapse^1,3^.

Long non-coding RNAs (lncRNAs) are dysregulated in cancer, leading to oncogenic or tumor-suppressive activities, as we reviewed^4^. Recent microarray studies have assessed the differential expression of lncRNAs among different subtypes of pediatric B-ALL^5,6^. However, the few studies so far on lncRNA expression and pediatric B-ALL patient survival were inconclusive or limited due to confounding variables or lack of statistical significance^6,7^.

In this work, we performed a comparative study of the lncRNA profiles of pediatric B-ALL patients with and without the ETV6-RUNX1 gene fusion and we combined our differential expression results with clinical information and external data. We obtained high-quality total RNA from 42 bone marrow samples from pediatric B-ALL patients and four bone marrows from healthy donors. Chromosomal abnormalities were assessed by a combination of karyotype analysis and gene fusion-specific polymerase chain reaction (PCR). LncRNA and messenger RNA (mRNA) expression analyses were performed using the LncPath™ Human Cancer Array (Arraystar Inc.), which includes 2829 lncRNAs and 1906 protein-coding genes. Because lncRNA functions are poorly defined, our pathway-focused approach allowed us to predict lncRNA functions based on the function of their associated mRNAs. A full description of our methods is included in the Supplementary Material. Our microarray data has been uploaded to Gene Expression Omnibus under the accession GSE128254.

Our pediatric B-ALL cohort included 24 ETV6-RUNX1-positive patients and 18 ETV6-RUNX1-negative patients (Supplementary Table S1). None of the ETV6-RUNX1-negative patients had any other known chromosomal rearrangements (7 had normal karyotype, 8 were hyperdiploid and 3 had unsuccessful karyotype analysis but no gene fusions were identified by PCR). There were no differences between the ETV6-RUNX1-positive and the ETV6-RUNX1-negative subgroups regarding patient sex, age, phenotype, central nervous system involvement, percentage of blasts or risk group (Supplementary Table S2). In addition, most patients were treated using similar therapy protocols based on Spanish PETHEMA and SEHOP protocols.

**Fig. 1:**
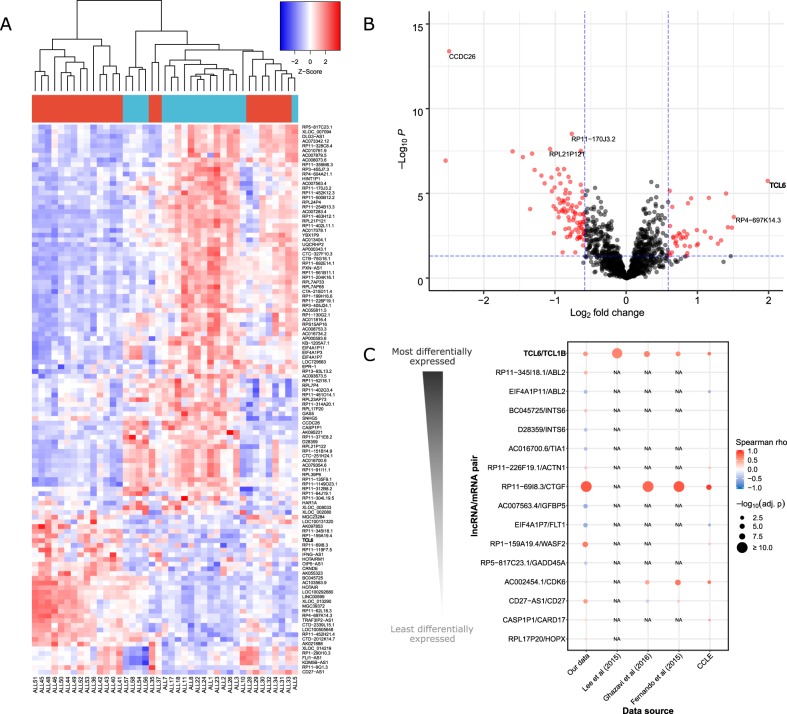
Differential expression of lncRNAs in ETV6-RUNX1-positive pediatric B-ALL vs. ETV6-RUNX1-negative pediatric B-ALL. **a** Heatmap of differentially expressed lncRNAs. On top of the heatmap, ETV6-RUNX1-negative B-ALL samples are labeled in blue and ETV6-RUNX1-positive B-ALL samples are labeled in red. Clustering was performed based on the Spearman correlation coefficient. Our candidate lncRNA, TCL6, is highlighted in bold. **b** Volcano plot of the differential expression results. The horizontal dashed line represents a threshold of FDR = 0.05. The vertical dashed line represents the thresholds of fold change = −1.5 and fold change = 1.5. Red dots represent the statistically significant differentially expressed lncRNAs. Our candidate lncRNA, TCL6, is highlighted in bold. **c** Correlation of the expression of lncRNA/mRNA pairs in pediatric B-ALL datasets. We focused our analysis on the unique lncRNA/mRNA pairs that were differentially expressed in our microarray data. For each pair, the Spearman correlation coefficient between the normalized expression of the lncRNA and the normalized expression of the associated mRNA was assessed in our data, as well as in external datasets of B-ALL patients and cell lines. Larger dots represent lower FDR-adjusted *p*-values. Our candidate lncRNA/mRNA pair, TCL6/TCL1B, is highlighted in bold. The sample sizes were: *n* = 46 for our data, *n* = 80 for Lee et al.^8^, *n* = 64 for Ghazavi et al.^5^ and *n* = 44 for Fernando et al.^6^. In addition, we analyzed data from 13 B-ALL cell lines from the Cancer Cell Line Encyclopedia (CCLE). Missing values are represented with an “NA” mark.

By performing an unsupervised hierarchical clustering based on lncRNA expression profiles, all healthy samples clustered together and most ETV6-RUNX1-positive samples were separated from most ETV6-RUNX1-negative samples (Supplementary Fig. S1). To provide insights into ETV6-RUNX1-specific lncRNA expression signatures, we compared lncRNA expression in ETV6-RUNX1-positive vs. ETV6-RUNX1-negative pediatric B-ALL. We identified 117 differentially expressed lncRNAs (Fig. 1a, b and Supplementary Table S3). The top upregulated lncRNA in the ETV6-RUNX1-positive subgroup was TCL6 (~3.8-fold) and the top down-regulated lncRNA was CCDC26 (~5.6-fold). Furthermore, we found no significant enrichment of differentially expressed lncRNAs or mRNAs from chromosomes 12 or 21, suggesting that the t(12;21) translocation affects gene expression at a genome-wide level. Using a Gene Ontology Analysis, we identified overrepresented cancer-related pathways among the differentially expressed lncRNAs/mRNAs in the ETV6-RUNX1-positive *vs*. ETV6-RUNX1-negative comparison (Supplementary Fig. S2 and Supplementary Table S4).

**Fig. 2:**
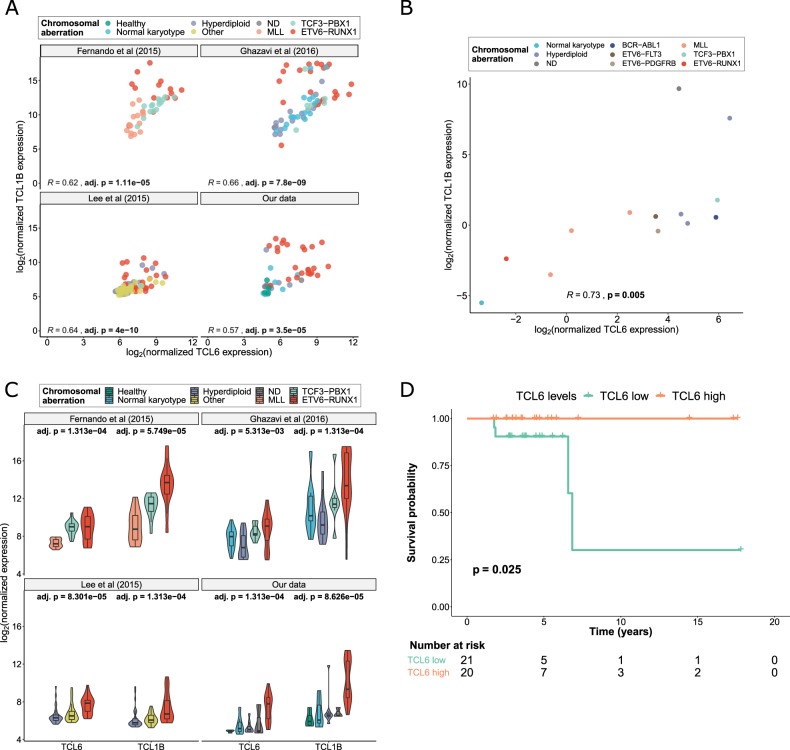
Validation of the relevance of TCL6/TCL1B in pediatric B-ALL. **a** Correlation analysis of TCL6/TCL1B in independent datasets of pediatric B-ALL patients. The points are colored according to the genomic aberration of the samples. The *p*-values were adjusted using the Benjamini–Hochberg method. Sample sizes: *n* = 46 for our data, *n* = 80 for Lee et al.^8^, *n* = 64 for Ghazavi et al.^5^, *n* = 44 for Fernando et al (2015). **b**. Correlation analysis of TCL6/TCL1B in the Cancer Cell Line Encyclopedia (CCLE), *n* = 13. The points are colored according to the genomic aberration of the samples. **c**. Validation of the differential expression of TCL6 and TCL1B in three independent external B-ALL cohorts. Sample sizes: *n* = 46 for our data, *n* = 80 for Lee et al (2015), *n* = 64 for Ghazavi et al (2016) and *n* = 44 for Fernando et al.^6^. Kruskal–Wallis FDR-adjusted *p*-values are shown. **d** Kaplan–Meier curve of pediatric B-ALL patients divided in two groups: “TCL6 high” and “TCL6 low”, based on whether TCL6 expression was above or beyond the median. The logrank *p*-value is shown.

Based on our predicted lncRNA/mRNA associations, we identified 19 pairs of lncRNA/mRNA probes that were differentially expressed between ETV6-RUNX1-positive and ETV6-RUNX1-negative pediatric B-ALL (Supplementary Fig. S3 and Supplementary Table S5). The top differentially expressed lncRNA/mRNA pair was TCL6/TCL1B. As a clue for putative co-regulation, we assessed the correlation of the expression of the lncRNA/mRNA pairs in our dataset as well as in three independent external microarray studies in pediatric B-ALL patients^5,6,8^ (combined *n* = 234; see Supplementary Table S6 for details on sample sizes) and in the Cancer Cell Line Encyclopedia. Only two lncRNA/mRNA pairs, TCL6/TCL1B and RP11-69I8.3/CTGF, were consistently correlated across all analyzed datasets (Figs. 1c, 2a, b). Based on genomic information, *TCL6* and *TCL1B* are likely to originate from different regulatory elements (Supplementary Note). Taken together, these results led us to further investigate TCL6/TCL1B.

We confirmed the differential expression of TCL6/TCL1B in a subset of our cohort using quantitative RT-PCR (Supplementary Fig. S4). In addition, we further validated the differential expression of TCL6/TCL1B among pediatric B-ALL subgroups using external data^5,6,8^. Both TCL6 and TCL1B were significantly upregulated in ETV6-RUNX1-positive pediatric B-ALL compared to hyperdiploid B-ALL, B-ALL with normal karyotype, and B-ALL bearing any translocation but TCF3-PBX1 (Fig. 2c and Supplementary Table S7). These results show that high TCL6 and TCL1B expression levels are strongly associated with ETV6-RUNX1-positive B-ALL.

Finally, we studied whether TCL6 or TCL1B expression could predict patient survival. We observed a statistically significant decrease in disease-free survival in “TCL6 low” patients when compared to “TCL6 high” (logrank *p* *=* 0.025, Fig. 2d), but we did not observe this trend for TCL1B. Remarkably, all four events (two deaths and two relapses) affected “TCL6 low” patients. The two relapses affected ETV6-RUNX1-positive patients and the two deaths affected ETV6-RUNX1-negative patients. Interestingly, the patients who relapsed (ALL35 and ALL36) had the second and fourth lowest TCL6 expression levels within the ETV6-RUNX1-positive subgroup. To validate our results, we found no external pediatric B-ALL datasets containing survival information and lncRNA expression. However, using Prognoscan^9^, we found that high TCL6 expression associates with better patient prognosis in multiple myeloma (GEO dataset GSE2658) and in one out of four acute myeloid leukemia datasets (GSE12417). Future research using larger patient cohorts should confirm the role of TCL6 in pediatric B-ALL and its usefulness as a prognostic biomarker.

TCL6 is thought to be involved in leukemogenesis, as well as in mature B-cell neoplasms, but it has never been shown to affect patient survival^10,11^. Although the molecular function of TCL6 in cancer is unknown, it may modulate the EGFR/AKT pathway at least in placental tissue^12^. Here, we have predicted a functional relationship between *TCL6* and its neighboring protein-coding gene, *TCL1B*. TCL1B is a co-activator of the protein kinase AKT1 in T-cell leukemias and it has oncogenic activity in vivo^13,14^. According to our results, TCL6 and TCL1B are highly associated with ETV6-RUNX1-positive pediatric B-ALL, in which the AKT pathway plays a major role^15^. Based on this information, we propose a functional link between TCL6, TCL1B and the AKT1 pathway, but further research should be performed to confirm this hypothesis.

In conclusion, we have identified TCL6 as a novel lncRNA strongly related to ETV6-RUNX1-positive pediatric B-ALL. We suggest that low TCL6 levels may be associated with poor disease-free survival, even within ETV6-RUNX1-positive B-ALL. LncRNA expression analysis could complement current cytogenetic and molecular biological analyses applied in the routine diagnosis of pediatric B-ALL to allow a better stratification of a larger number of patients into risk-based treatment groups.

## Supplementary information

Supplemental material

Figure S1

Figure S2

Figure S3

Figure S4
