# Supplementary material for: Expression of the long non-coding RNA TCL6 is associated with clinical outcome in pediatric B-cell acute lymphoblastic leukemia
Source: Blood Cancer J. 2019 Nov 25;9(12):93. doi: 10.1038/s41408-019-0258-9 (PMC6877621; doi:10.1038/s41408-019-0258-9)
Supplement: Supplementary file 1 — Supplemental material [file 41408_2019_258_MOESM1_ESM.docx]

Supplementary Material. Table of contents.

[Supplementary Methods 2](#_Toc20322330)

[Patients 2](#_Toc20322331)

[Diagnostic tests 2](#_Toc20322332)

[RNA extraction 2](#_Toc20322333)

[RNA labeling and array hybridization 2](#_Toc20322334)

[Microarray design and predicted regulation between lncRNAs and protein-coding genes 3](#_Toc20322335)

[Data pre-processing and differential expression analyses 3](#_Toc20322336)

[Data availability 4](#_Toc20322337)

[Quantitative RT-PCR analysis 4](#_Toc20322338)

[Analysis of external data 4](#_Toc20322339)

[Gene Ontology analyses 5](#_Toc20322340)

[Survival analyses 5](#_Toc20322341)

[Supplementary Note 7](#_Toc20322342)

[Genomic context of TCL6 and TCL1B 7](#_Toc20322343)

[Supplementary References 7](#_Toc20322344)

[Supplementary Tables 8](#_Toc20322345)

[Table S1. Clinical and demographic characteristics of the pediatric B-ALL patients. 8](#_Toc20322346)

[Table S2. Differences in clinical characteristics between ETV6-RUNX1-positive and ETV6-RUNX1-negative patients 10](#_Toc20322347)

[Table S3. 117 lncRNAs that were differentially expressed between ETV6-RUNX1-positive and ETV6-RUNX1-negative pediatric B-ALL. 11](#_Toc20322348)

[Table S4. Top 10 differentially expressed non-redundant Gene Ontology (GO) terms between ETV6-RUNX1-positive and ETV6-RUNX1-negative pediatric B-ALL. 16](#_Toc20322349)

[Table S5. Pairs of long non-coding RNAs (lncRNAs) and associated messenger RNAs (mRNAs) differentially expressed between ETV6-RUNX1-positive and ETV6-RUNX1-negative pediatric B-ALL. 18](#_Toc20322350)

[Table S6. Number of pediatric B-ALL patients in the internal and external cohorts used in our analyses. 19](#_Toc20322351)

[Table S7. Kruskal-Wallis and Wilcoxon rank-sum p values for the differential expression of TCL6 and TCL1B in all possible comparisons between pediatric B-ALL subtypes in internal and external cohorts. 20](#_Toc20322352)

[Supplementary figure legends. 21](#_Toc20322353)

# Supplementary Methods

Unless specified otherwise, all computational analyses were performed using R (version 3.6.0) and Bioconductor (version 3.9). Normality of quantitative data was assessed using quantile-quantile plots and the Shapiro-Wilk tests, and data transformations and statistical tests were chosen accordingly as described in each sub-section.

## Patients

Forty-two bone marrow samples of pediatric B-cell acute lymphoblastic leukemia (B-ALL) patients and four bone marrows from healthy donors were obtained. All patients provided written informed consent for the use of their samples and clinical data for research purposes in accordance with the Declaration of Helsinki. Bone marrow tissues were frozen upon acquisition and stored at −80°C. All sample handling, procedure and storage were the same for leukemic and control subjects. Clinical information was collected from medical reports (Supplementary Material. Table S1). Most of the cases were treated using similar therapy protocols based on Spanish PETHEMA and SEHOP protocols. The study was approved by the ethics committees of the Universitary Regional Hospital of Málaga (Málaga, Spain), Universitary Virgen de las Nieves Hospital (Granada, Spain), and San Juan de Dios Hospital (Barcelona, Spain).

## Diagnostic tests

The diagnosis of pediatric B-ALL and lineage discrimination was made by morphology and immunophenotypic analyses. Karyotype analyses were requested for all samples. In addition, the following gene fusions were assayed by polymerase chain reaction (PCR) in all samples: t(9;22)[BCR/ABL1], t(12;21)[ETV6/RUNX1], t(4;11)[MLL/AF4] and t(1;19)[TCF3/PBX1].

## RNA extraction

Total RNA was extracted from bone marrow aspirates using Trizol reagent (Invitrogen), according to manufacturer’s instructions. Quality of RNA samples was measured using an Agilent 2100 Bioanalyzer system. All RNA samples submitted to the microarray analysis were determined to be of high quality.

## RNA labeling and array hybridization

To amplify RNA, we used the Epicentre’s TargetAmp 1-Round aRNA Amplification kit (Ilumina). For microarray analysis, an Agilent Array platform was employed. The sample preparation and microarray hybridization were performed based on the manufacturer’s standard protocols. Briefly, total RNA from each sample was amplified and transcribed into fluorescent cRNA following Agilent’s Quick Amp Labeling protocol (version 5.7, Agilent Technologies). The labeled cRNAs were hybridized onto the LncPath^TM^ Human Cancer Array (8x15K, Arraystar). After washing the slides, the arrays were scanned by the Agilent Scanner G2505C. Agilent Feature Extraction software (version 11.0.1.1) was used to analyze the acquired array images.

## Microarray design and predicted regulation between lncRNAs and protein-coding genes

The LncPath^TM^ Human Cancer Array is designed to measure the expression of 2829 lncRNAs and 1906 protein-coding genes that have been selected based on the following criteria. The lncRNA list was extracted from GENCODE, RefSeq, GenBank, UCSC known genes and from the lncRNAs described by Cabili et al. (2011)^1^. The protein-coding genes were selected based on their predicted regulation by lncRNAs. Briefly, the lncRNAs selected for the analysis were classified based on their predicted interaction with coding genes:

1. Neighboring lncRNAs, defined as lncRNAs that locate within 3 kb away from critical cancer pathway genes. These lncRNAs are predicted to modulate the expression of neighboring protein-coding genes.
2. Competing endogenous RNAs (ceRNAs), which share miRNA response elements with the mRNA transcripts of critical pathway genes and, therefore, might prevent these mRNAs from being degraded. These lncRNAs are predicted to affect the expression of the protein-coding genes that are targeted by the related miRNAs.
3. Enhancer-like lncRNAs, which locate within 300 kb of critical cancer genes and might act as enhancers according to scientific publications.

## Data pre-processing and differential expression analyses

All subsequent analyses were performed using the R software’s limma package. First, we applied quantile normalization on the raw data. Low intensity filtering was performed, and the LncRNAs/Coding Genes for which at least 4 out of 46 samples had the “P” or “M” flags (“All Targets Value”) were retained for further analyses. Differentially expressed lncRNAs/mRNAs were identified by filtering by false discovery rate (FDR) < 0.05 and fold change (absolute value) > 1.5. Hierarchical clustering based on the Spearman correlation coefficient was performed to show lncRNA and mRNA expression patterns among samples.

## Data availability

The microarray data has been deposited on Gene Expression Omnibus under the accession GSE128254.

## Quantitative RT-PCR analysis

Quantitative RT-PCR reactions were performed using the SYBR Green reagent and the ABI PRISM 7500 system (Applied Biosystems) under the conditions recommended by the manufacturers. GAPDH was used as a reference gene after we confirmed that its expression was stable across the samples. The reactions were performed in triplicate. Expression was estimated using the 2^(−ΔΔCt)^ method and differential expression was assessed using Student’s t-tests on the log_2_-transformed data.

## Analysis of external data

In order to further confirm the differential expression patterns of our statistically significant lncRNA/mRNA pairs, we compared our results in B-ALL patients with those from previous microarray studies^2-4^. First, we downloaded the normalized, log2-transformed expression data from Gene Expression Omnibus using the R package “GEOquery” (dataset identifiers: GSE65645, GSE79868 and GSE56599). An exception was GSE79868, in which TCL6 expression data had been filtered out by the authors due to strict filtering criteria, and therefore the raw expression data was downloaded from GEO, normal-exponential background correction was performed using the ‘normexp’ method and quantile normalization between arrays was applied (R package ‘limma’). Then, we filtered the expression data to keep the probes that measured our lncRNAs or mRNAs of interest. Due to inconsistencies in lncRNA annotation, we searched for overlaps, at the genomic coordinate level, between the microarray probes and the exons of our genes of interest. For each external dataset, we compared the expression of each of our candidate lncRNAs and mRNAs among all available B-ALL subtypes that had more than one patient. We performed a Kruskal-Wallis test for the comparisons that involved more than two groups, as well as Wilcoxon rank-sum tests for all pairwise comparisons. In addition, we studied the correlation between the expression levels of each lncRNA/mRNA pair using the Spearman correlation coefficient. All p-values were corrected for multiple comparisons to control the false discovery rate (FDR) using the Benjamini-Hochberg method. A summary of the number of patients per B-ALL subtype in each study is included in Supplementary Material. Table S4.

In addition, in order to validate our results in ALL cell lines, we analyzed external gene expression data from the Cancer Cell Line Encyclopedia (CCLE). First, we downloaded the raw gene counts file (version 2018-09-29) and the cell line annotation file (version 2018-12-26) from the CCLE website (<https://portals.broadinstitute.org/ccle/data>). We identified all B-ALL cell lines by searching for “type_refined” == “B-cell ALL” in the cell line annotation file. Out of the 20 identified B-ALL cell lines, 13 had available gene expression data as well as karyotype information in the American Type Culture Collection (ATCC), in the German Collection of Microorganisms and Cell Cultures (DSMZ), or in the publication that originally described the cell line. Gene expression counts were loaded into R (package ‘edgeR’) and the data were filtered to include the 13 B-ALL cell lines of interest. Read counts were normalized using the trimmed mean of M-values method and they were transformed to log2-counts per million using cpm(<tmm_normalized_counts>, log = TRUE). Then, the transformed data were filtered to only include the lncRNAs and mRNAs of interest. Spearman correlation coefficients for the expression of each lncRNA/mRNA pair were calculated.

## Gene Ontology analyses

We performed Gene Ontology analyses to identify significantly enriched biological pathways, molecular functions or cellular components related to the differentially expressed lncRNAs identified in the microarray analyses (FDR < 0.05, fold change (absolute value) > 1.5). First, we identified the protein-coding genes associated to the differentially expressed lncRNAs. Then, we used Enrichr^5^ to identify significantly enriched Gene Ontology terms. We analyzed the up-regulated and the down-regulated genes separately. In order to remove redundancies in Gene Ontology terms, we collapsed related terms using ReviGO^6^ with default parameters and providing the FDR values for each GO term.

## Survival analyses

To assess whether TCL6 expression levels correlated with patient prognosis, we performed a survival analysis using the packages ‘survival’ and ‘survminer’ in R software. Both deaths and relapses were considered as events. We split the patients in two groups (“TCL6 high” or “TCL6 low”) based on whether their normalized and log_2_-transformed TCL6 expression was above or below the median. We plotted Kaplan-Meier curves for “TCL6 high” and “TCL6 low” patients and compared both groups using a univariate logrank test. We performed the same analysis for TCL1B expression.

# Supplementary Note

## Genomic context of TCL6 and TCL1B

Because a significant correlation between lncRNA and mRNA expression levels does not necessarily mean that the lncRNA regulates its associated mRNA, we further studied the genomic location of the *TCL6* and *TCL1B* genes and we searched for nearby promoters and regulatory regions. *TCL6* and *TCL1B* are located in the same strand in chromosome 14 (q32.13) and *TCL1B* is ~13 kb downstream from the 3’ end of *TCL6*. According to GeneHancer^7^, which integrates promoter and enhancer data from various databases, *TCL1B* has a low-confidence promoter independent from *TCL6* (GeneHancer ID GH14J095686). However, *TCL6* has no clearly defined promoter in GeneHancer data and, instead, it is transcribed from a *TCL1B* enhancer element (GeneHancer ID GH14J095660). In Ensembl (release 96), the ENSR00000506415 promoter overlaps the *TCL6* transcription start site but only the ENSR00000072674 promoter flanking region is defined within ~2 kb of the transcription start site of *TCL1B*. Taken together, these results point to the possibility that *TCL6* and *TCL1B* may originate from different promoters. However, it cannot be ruled out that the *TCL6* promoter may be used for *TCL1B* transcription or that a common transcription factor may modulate both *TCL6* and *TCL1B*, which could explain the positive correlation between their expression levels.

# Supplementary References

1. Cabili, M.N. et al. Integrative annotation of human large intergenic noncoding RNAs reveals global properties and specific subclasses. *Genes Dev* **25**, 1915-1927 (2011).

2. Ghazavi, F. et al. Unique long non-coding RNA expression signature in ETV6/RUNX1-driven B-cell precursor acute lymphoblastic leukemia. *Oncotarget* **7**, 73769-73780 (2016).

3. Lee, S.T. et al. Epigenetic remodeling in B-cell acute lymphoblastic leukemia occurs in two tracks and employs embryonic stem cell-like signatures. *Nucleic Acids Res* **43**, 2590-2602 (2015).

4. Fernando, T.R. et al. LncRNA Expression Discriminates Karyotype and Predicts Survival in B-Lymphoblastic Leukemia. *Mol Cancer Res* **13**, 839-851 (2015).

5. Chen, E.Y. et al. Enrichr: interactive and collaborative HTML5 gene list enrichment analysis tool. *BMC Bioinformatics* **14**, 128 (2013).

6. Supek, F., Bosnjak, M., Skunca, N. & Smuc, T. REVIGO summarizes and visualizes long lists of gene ontology terms. *PLoS One* **6**, e21800 (2011).

7. Fishilevich, S. et al. GeneHancer: genome-wide integration of enhancers and target genes in GeneCards. *Database (Oxford)* **2017** (2017).

# Supplementary Tables

## Table S1. Clinical and demographic characteristics of the pediatric B-ALL patients.

| **Sample** | **t(12;21)** | **Sex** | **Age at diagnosis (years)** | **Phenotype** | **CNS**  **involvement** | **Dead or relapsed** | **Percentage of blasts** | **Karyotype** | **Risk group** | **Treatment** |
| --- | --- | --- | --- | --- | --- | --- | --- | --- | --- | --- |
| ALL1 | NO | F | 4.71 | B common | 1 | 0 | 98 | 55XX dup(1)+4, +6, +10, +14, del 16, +17, +18, +21, +21 | Intermediate | RI LAL/SEHOP-PETHEMA 2013 |
| ALL2 | NO | F | 6.37 | B common | traumatic | 0 | 95 | 46XX | Intermediate | RI LAL/SEHOP-PETHEMA 2013 |
| ALL3 | NO | M | 6.27 | B common | 1 | 0 | 90 | 52XY,+X,+Y,+17,+18,+21,+21[10]/46XY[10] | Intermediate | RI LAL/SEHOP-PETHEMA 2013 |
| ALL5 | NO | M | 3.64 | B common | 1 | 0 | 92 | 46XY | Intermediate | RI LAL/SEHOP-PETHEMA 2013 |
| ALL7 | NO | M | 11.74 | B common | 3 | 0 | 98 | NA | Intermediate | RI LAL/SEHOP-PETHEMA 2013 |
| ALL8 | NO | M | 1.53 | Pre-B | 1 | 0 | 95 | 56XY,+4,+5,+6,+10,+11,+14,+17,+18,+21,+22[5]/46XY,[15] | High | AR LAL/SEHOP-PETHEMA 2013 |
| ALL10 | NO | M | 6.86 | B common | 1 | 0 | 92 | 46XY | Intermediate | RI LAL/SEHOP-PETHEMA 2013 |
| ALL11 | NO | F | 12.80 | B common | 1 | 0 | 80 | 46XX | Intermediate | RI LAL/SEHOP-PETHEMA 2013 |
| ALL17 | NO | M | 2.89 | B common | 1 | 0 | 93 | 56XY,+X,+Y,+4,+6,+10,+14,+17,+18,+21,+21[11]/46XY[9] | Low | SEHOP PETHEMA 2013 |
| ALL18 | NO | F | 7.47 | B common | 1 | 0 | 80 | 46XX | Low | PETHEMA BR-01 |
| ALL22 | NO | F | 11.35 | B common | 1 | 0 | 90 | 46XX | High | PETHEMA AR-05 |
| ALL23 | NO | NA | NA | NA | NA | NA | NA | NA | NA | NA |
| ALL24 | NO | M | 2.61 | B common | 1 | 1 | 92 | NA | Intermediate | PETHEMA RIA-96 |
| ALL26 | NO | M | 0.62 | B common | 3 | 1 | 95 | 47,XY,+8[7]/46,XY[13] | High | SHOP-baby |
| ALL28 | YES | F | 5.04 | B common | 1 | 0 | 97 | NA | High | SEHOP/PETHEMA2013 |
| ALL29 | YES | F | 2.37 | B common | 1 | 0 | 95 | 46XX | Intermediate | RI LAL/SEHOP-PETHEMA 2013 |
| ALL30 | YES | F | 4.08 | B common | 1 | 0 | 98 | NA | High | AR-SEHOP/PETHEMA2013 |
| ALL31 | YES | M | 8.37 | B common | 1 | 0 | 98 | 48XY,del(6)(q22q24),der(11)t(11;17)(q13;q21),+16,+21[8]/46XY[12] | Low | SEHOP-PETHEMA 2013 standard risk |
| ALL32 | YES | M | 3.11 | B common | 1 | 0 | 90 | NA | Intermediate | PETHEMA standard risk |
| ALL33 | YES | M | 2.59 | B common | 1 | 0 | 98 | NA | Intermediate | PETHEMA RIA-96 |
| ALL34 | YES | F | 9.01 | B common | 1 | 0 | 98 | 46XX | Low | PETHEMA BR/01 |
| ALL35 | YES | M | 9.47 | B common | 1 | 1 | 67 | 46XY | Low | PETHEMA BR/01 |
| ALL36 | YES | M | 6.14 | B common | 1 | 1 | 98 | 46XY | Low | PETHEMA BR/01 |
|  |  |  |  |  |  |  |  |  |  |  |
| **Sample** | **t(12;21)** | **Sex** | **Age at diagnosis (years)** | **Phenotype** | **CNS**  **involvement** | **Dead or relapsed** | **Percentage of blasts** | **Karyotype** | **Risk group** | **Treatment** |
| ALL37 | YES | M | 2.57 | B common | 1 | 0 | 90 | 46XY | Intermediate | PETHEMA RIA-96 |
| ALL40 | YES | M | 11.64 | B common | 1 | 0 | 96 | 86 XXXY, -1,-1,-2,-2,-5,+6,-8,-10,+11,-12,-12,-15,+16,-22,-22,+3mar in 3 metaf. 46XY in 17 metaf. | High | PETHEMA RIA-96 |
| ALL41 | YES | M | 3.09 | B common | 1 | 0 | 92 | 46XY | Intermediate | PETHEMA RIA-96 |
| ALL42 | YES | M | 4.95 | Pre-B | 1 | 0 | 98 | 46XY | High | PETHEMA PROTOCOL 27/89 |
| ALL43 | YES | M | 8.87 | Pre-B | 1 | 0 | 90 | NA | Low | PETHEMA 27/89 |
| ALL44 |  | F | 11.20 | B common | 1 | 0 | 100 | 46,XX,?t(7;9),?t(9;12),t(9;22)(q34;q11)[22]/46,XX [3] | Very high | SEHOP-PETHEMA 2013 |
| ALL45 | YES | F | 3.32 | B common | traumatic | 0 | 93 | 46,XX[20] | Intermediate | SEHOP-PETHEMA 2013 |
| ALL46 | YES | M | 5.33 | B common | 1 | 0 | 84 | 48,XY,del(6)(q?13q26-27),+mar1,+mar2[4]/47,XY,del(6)(q?13q26-27+mar1[4]/46,XY[16] | Intermediate | SEHOP-PETHEMA 2013 |
| ALL47 | YES | F | 5.02 | B common | 1 | 0 | 99 | 46,XX,der(3)(2)/46,XX,del(12)(p13)(4)/46,XX(4) | Intermediate | SEHOP-PETHEMA 2013 |
| ALL48 | YES | F | 4.33 | B common | 1 | 0 | 90 | 46,XX[20] | Intermediate | SEHOP-PETHEMA 2013 |
| ALL49 | YES | F | 3.67 | B common | 1 | 0 | 96 | 46,XX [20] | Intermediate | SHOP-2005 |
| ALL50 | YES | F | 2.89 | B common | 1 | 0 | 99 | 46,XX[20] | Intermediate | SHOP-2005 |
| ALL51 | YES | M | 11.65 | B common | 1 | 0 | 93 | 46,XY[10] | Intermediate | SHOP-2005 |
| ALL52 | YES | M | 4.66 | B common | 1 | 0 | 96 | 47,XX,t(12;21)(p13;q22),+der(21)/ t(12;21)(p13;q22)(6)/46,XX(17) | Intermediate | SHOP-2005 |
| ALL53 | YES | M | 8.96 | B common | 1 | 0 | 93 | 48,XY,+mar1,+mar2[3]/46,XY[42] | Intermediate | SEHOP-PETHEMA 2013 |
| ALL54 | NO | F | 6.14 | B common | 1 | 0 | 93 | 52,XXX,+5,+17,+18,+21,+22[4]/46,XX[16] | Intermediate | SEHOP-PETHEMA 2013 |
| ALL56 | NO | M | 3.75 | B common | traumatic | 0 | 100 | 71 chromosomes in mosaic | Intermediate | SEHOP-PETHEMA 2013 |
| ALL57 | NO | F | 10.17 | B common | 1 | 0 | 81 | 56,XX,+X,+X,+4,+5,?del(6)(q),+8,+9,+14,+18,+21,+22(10)/46,XX(20) | Intermediate | SEHOP-PETHEMA 2013 |
| ALL58 | NO | F | 2.96 | B common | 1 | 0 | 98 | 46,XX [30] | Intermediate | SEHOP-PETHEMA 2013 |

CNS: central nervous system; CNS 1 means no CNS involvement, CNS 2 is a very low level, and CNS 3 is definite CNS involvement. F: female*;* NA: not available; M: male; MRD: minimal residual disease

## **Table S2. Differences in clinical characteristics between ETV6-RUNX1-positive and ETV6-RUNX1-negative patients**. Differences were assessed by fitting a logistic regression model where the dependent variable was the presence or absence of the ETV6-RUNX1 translocation and the independent variables were all the relevant clinical characteristics of the patients. The p values of the regression coefficients for each dependent variable are reported.

| **Clinical characteristic** | **p** |
| --- | --- |
| Sex | 0.681 |
| Phenotype | 0.982 |
| Age at diagnosis | 0.634 |
| CNS involvement | 0.100 |
| Percentage of blasts | 0.338 |
| Risk (intermediate vs low) | 0.444 |
| Risk (intermediate vs high) | 0.364 |

## **Table S3. 117 lncRNAs that were differentially expressed between ETV6-RUNX1-positive and ETV6-RUNX1-negative pediatric B-ALL.** Overexpressed lncRNAs are in red and downregulated lncRNAs are in green. The lncRNAs are ranked by fold change.

| **GeneName** | **FC** | **P-value** | **FDR** | **seqname** | **source** | **Probe sequence** |
| --- | --- | --- | --- | --- | --- | --- |
| TCL6 | 3.8006201 | 1.37E-05 | 0.000776438 | NR_028288 | RefSeq | CATGGAACATCTGCTCTGTGCTTATATCTCATATGCATTATCTCTA |
| RP4-697K14.3 | 2.6994943 | 0.000863057 | 0.010626896 | ENST00000458368 | gencode | GTGAGTGTGGAGTAGGCATTATTGTACATTGTAGTCCTTTCTCCTC |
| LOC100292680 | 2.6693924 | 0.001914082 | 0.017727121 | NR_028415 | RefSeq | GTGTGTATGTAGGTGTCTCTGTAGACAGTGATTAAAAATACTCCTG |
| RP11-345I18.1 | 2.6189587 | 4.76E-05 | 0.001758135 | ENST00000424885 | gencode | GGACCGGGTGCCTTACATCCACAGCAAGTTTAAGAAGGATGATTGA |
| LINC00599 | 2.5739652 | 0.00121065 | 0.013516529 | NR_024281 | RefSeq | CGGAGTGCTTGTGTGTGCAGATTTTTCTCTGGGCTCAGGACTCATT |
| TRAF3IP2-AS1 | 2.5719387 | 2.94E-05 | 0.001325599 | NR_034111 | RefSeq | GTGGTGGTGCCTGTCTGGTTTTAGTTGTTTCTTTTATCTTACACAG |
| HOTAIR | 2.4027148 | 0.0063445 | 0.037648951 | NR_003716 | RefSeq | CTTGTGTAGGTTGTGTGTGTGTGGTGGTTTTATGCATAAATAAAGT |
| KDM5B-AS1 | 2.1692214 | 0.001319535 | 0.014226813 | NR_046325 | RefSeq | TCTCCCCCAGTCTCCAGTGCACCGGCTTTCCCTCGTCCTCTGCGCA |
| LOC100131320 | 2.1419517 | 9.63E-05 | 0.002777045 | NR_036537 | RefSeq | CTCCGAAGAGAAATAATGATAGTAATAGTGGTGCTGGGAACAATAT |
| AC103563.9 | 2.0721975 | 0.005082797 | 0.033112075 | ENST00000442200 | gencode | CCTGTCTGCGTGAACATATTCGTGTCTATCCAACTCAACTAAAAAA |
| FLI1-AS1 | 1.9703387 | 0.007445759 | 0.042064418 | NR_038908 | RefSeq | CCTCCCCCACTGGAATTATTCAATATGGAACTAGATTAAATTGAAG |
| CRNDE | 1.9345423 | 0.000845668 | 0.010612438 | NR_110454 | RefSeq | CTGAAGATAAGGAGTTCTCTTGTAGGATGCCACTGGAAATGTTGAA |
| BC045725 | 1.9287731 | 0.004162493 | 0.029367632 | uc001vfn.3 | UCSC_knowngene | GTACTTGCTGAGGATGTACTGTGTATTAGGTGTGGTGCATATAAAA |
| IFNG-AS1 | 1.8253476 | 9.47E-05 | 0.002777045 | NR_104124 | RefSeq | CCATACTGATCATCCTCAGCAATCATGTGAGTTATTAGTCTAAGTA |
| RP11-69I8.3 | 1.7928616 | 0.005668426 | 0.035713419 | ENST00000435287 | gencode | GTAACTTGGACAACCTGAGGCATTAATTGAGGATCAATATGATGAC |
| LOC100505648 | 1.7326862 | 0.007183344 | 0.041189884 | NR_040058 | RefSeq | TGCCGAAACTGGAAGGTTACATTTAGTTCAGGATATGAAGATCATA |
| MGC39372 | 1.7307408 | 0.009445403 | 0.047785669 | NR_033851 | RefSeq | GCGTGCCTGGGTGGAAAATGAGCTTATATATGAAGAAGTCAGCGAG |
| RP11-119F7.5 | 1.7289208 | 0.000115562 | 0.003080095 | ENST00000562082 | gencode | GCAAATTGTATCATTCCTGATTACACAGAACTTTGTGGGTGGTTTT |
| RP1-290I10.3 | 1.7025559 | 0.009457255 | 0.047785669 | ENST00000420389 | gencode | ACCTTCTCCCTGGACACCTTTGCTAAGAGATTTGGCTAATTTCTTC |
| RP1-159A19.4 | 1.6659938 | 0.006082385 | 0.036620293 | ENST00000443579 | gencode | GGTTTGAATGTACTCACTGAGTGGTCTTCATAAATTACCTCTCTGC |
| XLOC_013290 | 1.6544924 | 0.009776633 | 0.048601824 | TCONS_00027288 | Cabili et al | ATGTCTGGGTGTCAGGTGTCTGGGTGTCAGGTGTCTCAGTATCTGG |
| AK055323 | 1.634434 | 0.010345285 | 0.048884665 | uc003fdu.3 | UCSC_knowngene | CATGCTATCTCTGATCCTGTGGTGTTGTATAAAAATGAACAGCTAA |
| RP11-62L18.3 | 1.6308012 | 0.005263474 | 0.033686245 | ENST00000434988 | gencode | ACTGCTCTGTGTCTGCACGTGTTCGCATTTATTAAATGTGGAGGTG |
| CTD-2339L15.1 | 1.6290913 | 0.000566859 | 0.007818575 | ENST00000526635 | gencode | TGTGGTTTGTTTCGGCAGTATCTGAATGTCAGCTTTTGGCACTTCT |
| XLOC_014219 | 1.6213989 | 0.002995861 | 0.023765091 | TCONS_00029585 | Cabili et al | CTCCTCTTCCCTCCTCAGCTCTCCCTCCTGTCAGGACAAGCAAAAG |
| MGC23284 | 1.5882592 | 0.000182104 | 0.003872237 | NR_024399 | RefSeq | CAGACATGGTTATGGGAAGTTTAATAAAACCGGTGAATCACGTGAA |
|  |  |  |  |  |  |  |
|  |  |  |  |  |  |  |
|  |  |  |  |  |  |  |
|  |  |  |  |  |  |  |
| **GeneName** | **FC** | **P-value** | **FDR** | **seqname** | **source** | **Probe sequence** |
| OIP5-AS1 | 1.5848481 | 0.007279371 | 0.041584765 | NR_026757 | RefSeq | CAGCAGAGGACCATTGTATGTATTGTCAGGTCTTTATATAAGAGTG |
| CD27-AS1 | 1.5659146 | 1.24E-05 | 0.000762211 | ENST00000545339 | gencode | CTGCCAGTCTCGAAAAGGCACTCTGTCACGTGTACACAGGAAAGGG |
| CTD-2012K14.7 | 1.5628572 | 0.000164599 | 0.003682421 | ENST00000562846 | gencode | GACCGTTCCGTTGTGAGAATGCTGGCCCAATAACATACTCTTTTTT |
| RP11-452H21.4 | 1.5396175 | 0.008772101 | 0.045680569 | ENST00000513207 | gencode | GCAAAAAGAAGTGGAACTGACTAGTGAGATGGTGGTGAGATTTTCT |
| HOTAIRM1 | 1.5342949 | 0.009235756 | 0.047290776 | NR_038367 | RefSeq | AAGATGAACTGGCGAGAGGTCTGTTTTGCCTGAACCCATCAACAGC |
| AK097853 | 1.5247401 | 0.001918475 | 0.017727121 | uc003owl.1 | UCSC_knowngene | GGGGTCTGGAAAAACAACAGGAATCTTGGGAATTATAAGGCTGAGA |
| RP11-9G1.3 | 1.5244921 | 1.12E-05 | 0.000734113 | ENST00000509715 | gencode | GCCATGTTCTCTTAATTCATCAGAGACTGAAAGCAAAGGAAAATCA |
| AK021888 | 1.5058815 | 6.21E-05 | 0.002159184 | uc003kor.1 | UCSC_knowngene | TGGCAGTCTTTCCCACCTCATTGGTCCGTGCTTTTATTTTTAAACC |

| **GeneName** | **FC** | **P-value** | **FDR** | **seqname** | **source** | **Probe sequence** |  |  |  |  |  |
| --- | --- | --- | --- | --- | --- | --- | --- | --- | --- | --- | --- |
| CCDC26 | -5.6150168 | 0 | 0 | NR_130917 | RefSeq | CTACCTAAAATCCCTAGAATAATCTCCATTTGGCTATGATGTGTTG |  |  |  |  |  |
| AC007283.4 | -5.6130505 | 4.17E-07 | 0.000078763 | ENST00000424739 | gencode | ATACCTTCAGAGAAAGAGAAGGCAGAGAAACTGAAGCAGTCCTTGT |  |  |  |  |  |
| RP11-463H12.1 | -2.986972 | 1.30E-07 | 0.000039745 | ENST00000484463 | gencode | CCATGCAACAGATTCCTTTATACCCTGATCATCACTGACAAAGAGA |  |  |  |  |  |
| AK095221 | -2.7202873 | 6.53E-07 | 0.000100043 | uc021uqd.1 | UCSC_knowngene | TATGACTGATACTGGCTACACAAGGCTCTAGGGCATCAGAAACATA |  |  |  |  |  |
| RP11-561B11.1 | -2.5398136 | 0.000285645 | 0.005026687 | ENST00000459899 | gencode | CAACTAAGTTTGAAAACGAGGAGTCGGAGGAGTTGGATGAGGATAT |  |  |  |  |  |
| RP11-135F9.1 | -2.5123493 | 1.14E-07 | 0.000039745 | ENST00000482189 | gencode | TTTCTTTTTCCTGAGGAAAGGGTACCCACCTCACCATTGTTGAAGA |  |  |  |  |  |
| RP11-371E8.2 | -2.4651718 | 2.34E-06 | 0.000255499 | ENST00000553543 | gencode | ACAAGCTTCGGAAACTCTTATCACTGCTGTTGATTCTAGCTCCAGT |  |  |  |  |  |
| RP1-151B14.9 | -2.280276 | 3.68E-06 | 0.00037611 | ENST00000436709 | gencode | ACAGCTCCAAAAGGAGATATTGGAAAGAACCAAGCTGGGTCTATAA |  |  |  |  |  |
| CTC-251H24.1 | -2.2080737 | 7.21E-06 | 0.000626465 | ENST00000467018 | gencode | TACAACTCCAAAAGGACACTGGAGAAGAACCAAGCTGGGTCTATAA |  |  |  |  |  |
| RP11-204K16.1 | -2.1734984 | 0.000185678 | 0.003894156 | ENST00000473054 | gencode | CTCGAAAAGGCAAAGGTTAAAGAACTTTGCCACTGAACTGGGTTAA |  |  |  |  |  |
| RPL21P121 | -2.0888103 | 1.03E-07 | 0.000039745 | ENST00000439258 | gencode | TTCCATTGAGGGAAGTACACTTTGTGAGAACCAATGGAAAAGAGCC |  |  |  |  |  |
| RP11-402L11.1 | -2.0412182 | 3.05E-05 | 0.00133336 | ENST00000475925 | gencode | GGCTGATATCCTTAGAAGCATGCCAGAACAGATGGGTGAAAAGTAA |  |  |  |  |  |
| RP11-500B12.2 | -2.0355474 | 0.000148547 | 0.003553532 | ENST00000436855 | gencode | GCCATTGGATGGAGAATTCATGTGATGCTGTACCCTTCAAGGATTT |  |  |  |  |  |
| HAR1A | -2.0336184 | 0.004678766 | 0.031695533 | NR_003244 | RefSeq | AAGTTTAGCATCCAATGACCCATTTAAGAGGTTTGCTGAGCCACGT |  |  |  |  |  |
| EIF4A1P11 | -2.0330359 | 7.23E-07 | 0.000100656 | ENST00000451239 | gencode | TCTTTGAGACATCAAGACCCTCTACAACACCTCCATTGAGGAAATG |  |  |  |  |  |
| AC017078.1 | -2.0058987 | 1.31E-05 | 0.000773435 | ENST00000416146 | gencode | GAGATCTTTGAACGGAAAGCCACATCTCACCAAGTAGGAAAAGAGA |  |  |  |  |  |
| AP000343.1 | -1.9505915 | 0.00022156 | 0.004259556 | ENST00000440602 | gencode | TCTGCTGGTACCTTACCAATGAGGATATCCAGTATCTCCGTGATTA |  |  |  |  |  |
| AP000593.6 | -1.9240862 | 1.36E-06 | 0.000160028 | ENST00000393668 | gencode | AGCTTCTGCAACTTCATGCATTTGAAGCTCATTTCCAGAGAGCTGA |  |  |  |  |  |
| AC079354.6 | -1.9228705 | 7.26E-05 | 0.00246062 | ENST00000453523 | gencode | AATCAGGTAAAACTCCAAAAGGAGCCATTGGAGAAGAACCAAGCTG |  |  |  |  |  |
| CTB-75G16.1 | -1.9091535 | 0.000717561 | 0.00938962 | ENST00000486628 | gencode | TTCATCAAGTCTTCCTGTCAGGAATTCACTGACCATCTCGTCAAGA |  |  |  |  |  |
| D28359 | -1.9079759 | 7.51E-06 | 0.000626465 | uc001vfo.1 | UCSC_knowngene | GCCTAACGCAGCCATGGCTTGTGGTCCCAAGAAGCATCTGAAGTGA |  |  |  |  |  |
| LOC729683 | -1.8893986 | 0.00045329 | 0.006609405 | NR_046273 | RefSeq | GAGACAGATCTGTTACAGGTTTTCAAATAAACTGGTCTATCTAGTG |  |  |  |  |  |
| EIF4A1P3 | -1.8893547 | 4.11E-07 | 0.000078763 | ENST00000411521 | gencode | GAGAACTTCTACAACACTTCCATTGAGGAAATTCCCCTCAATGTTC |  |  |  |  |  |
| AC016700.6 | -1.8676357 | 4.82E-05 | 0.001758135 | ENST00000446011 | gencode | AATCAGGTACAACTCCAAAAGGAGACATTGGAGAAGAACCAAGCTG |  |  |  |  |  |
| RP1-199H16.6 | -1.8640487 | 0.000136701 | 0.00334652 | ENST00000446147 | gencode | TGTGTGCTGCCAGTCAGTACGCGAAGGATATAGGTTTCATTAAGTT |  |  |  |  |  |
| CTC-327F10.3 | -1.8506254 | 0.000361675 | 0.005703225 | ENST00000509352 | gencode | ACTCCGAGTCATATAAAAGGCAGAAACTGGATCCTGGAAGCAAAGA |  |  |  |  |  |
| DLG3-AS1 | -1.844155 | 0.000613594 | 0.008240459 | ENST00000424211 | gencode | GCCCTAGGGGACAAAGGGGAGGAGGAGGCGAAAGAAGTCGGGAATG |  |  |  |  |  |
| RP11-52I18.1 | -1.8359161 | 4.58E-05 | 0.001758135 | ENST00000424289 | gencode | ATGAGACCAAACACAGACTTTCAAGACATGGCCTGTATGAGAGAAA |  |  |  |  |  |
| RPL7AP33 | -1.8287809 | 0.000210666 | 0.00422259 | ENST00000521552 | gencode | TGTGGTTCACATCGCCAAGCTTGAAAAGGCAAAGGCTAAAGAACTT |  |  |  |  |  |
| RP11-1149O23.1 | -1.8251113 | 4.63E-07 | 0.000078763 | ENST00000498163 | gencode | AGAAGCTCTATGATGTTTATGTGGCCAAGGTTCAACACCCTGATTA |  |  |  |  |  |
| AC008753.3 | -1.8184768 | 0.000115547 | 0.003080095 | ENST00000423096 | gencode | ATTGAACCTTTTTGACCTGGCTTGTTTGCAAAGGCCTGGTCAATGT |  |  |  |  |  |
|  |  |  |  |  |  |  |  |  |  |  |  |
| **GeneName** | **FC** | **P-value** | **FDR** | **seqname** | **source** | **Probe sequence** |  |  |  |  |  |
| RP11-226F19.1 | -1.8171047 | 0.000379119 | 0.005804315 | ENST00000465274 | gencode | CTGCCAGTGTTTCTGTCAGTCTGCAAAGGATATAGGTTTCATTAAG |  |  |  |  |  |
| AC073342.12 | -1.8122824 | 0.002941181 | 0.023452851 | ENST00000427392 | gencode | AATGTGAGAAAGAAATAAGCAACGGGGGTGATGTCCGTCTTTATGC |  |  |  |  |  |
| XLOC_008033 | -1.8092924 | 0.000294841 | 0.005103978 | TCONS_00017441 | Cabili et al | GGGGTAGGGGAAGAGCAAGTGAAGGATGCAGATTAATTTCTATTTA |  |  |  |  |  |
| HINT1P1 | -1.8029475 | 4.68E-05 | 0.001758135 | ENST00000464866 | gencode | CAGAAGATAATGACGAAAGTCTTCTTGGACACTTAATGATTGTTGG |  |  |  |  |  |
| RPS15AP16 | -1.7960419 | 0.000211103 | 0.00422259 | ENST00000472521 | gencode | AATAGTTTGGTTTCAAAGTGAGGTTGTACTGACAACCTCAGCTGGC |  |  |  |  |  |
| GAS5 | -1.7806229 | 0.000520146 | 0.007293856 | NR_002578 | RefSeq | CTGTCATACCTTTTAAAGGTATGGAGAGTCGGCTTGACTACACTGT |  |  |  |  |  |
| RP11-328C8.4 | -1.7654709 | 0.000368791 | 0.005703225 | ENST00000547824 | gencode | AAATAAATAAAGATTATGGGTATGGGGGAGCTGTGTGGGTGACACA |  |  |  |  |  |
| AC007563.4 | -1.7627526 | 2.55E-05 | 0.001248653 | ENST00000433233 | gencode | GGAATATCCATACTGTATCTGTGTGTGGTTGTCCAGAAAACATAAT |  |  |  |  |  |
| RP3-455J7.3 | -1.7600969 | 7.59E-05 | 0.0024719 | ENST00000422817 | gencode | ATGGAAACTATAGCTAGGTCCTGGTCTGGAAAACAAGCTCTGTGAA |  |  |  |  |  |
| RPL39P6 | -1.7537888 | 0.000176383 | 0.003857749 | ENST00000448289 | gencode | GGATTCGGATGAAAACTGGTAATAAAACCAGGTACAACTCCAAAAA |  |  |  |  |  |
| EIF4A1P7 | -1.732357 | 8.52E-07 | 0.000108706 | ENST00000421800 | gencode | GAAGACAAGAGGACTCTTTGAGACATCGAGACCTTCTATAATACCT |  |  |  |  |  |
| RP11-91I11.1 | -1.7322425 | 3.90E-05 | 0.001624173 | ENST00000439897 | gencode | CCTGTTCCCCAGTGGATTCAGATGAAAACTGGTAATAAAATCAGGT |  |  |  |  |  |
| RP11-304L19.5 | -1.7321298 | 0.005115239 | 0.033112075 | ENST00000563192 | gencode | TCTATTGAAGTGCTTAGCAATAAAGAAAGGTAGTGAGTTGATTCGG |  |  |  |  |  |
| PXN-AS1 | -1.7320085 | 0.002823579 | 0.022752106 | NR_038924 | RefSeq | GAGGAGCTTGCTCTGAGCTGCTGCCACCTGCTGGGCTGGAGCTGGA |  |  |  |  |  |
| AC093673.5 | -1.7311209 | 0.009661902 | 0.048601824 | ENST00000429630 | gencode | CTCCATCTGTAAACGTCACGGTTAATCCATCTACTTTATTGCATTA |  |  |  |  |  |
| KB-1205A7.1 | -1.7219822 | 8.18E-06 | 0.000626465 | ENST00000520175 | gencode | GCAGACATGAATACATTCCCTAACTTCAAATTTGAAGATCCCAAAC |  |  |  |  |  |
| RP11-170J3.2 | -1.7049296 | 9.44E-09 | 0.000007226 | ENST00000440453 | gencode | ATCAAATAGGCTGTGAAGAAGCTCTATGACACTGATGTGACCAAGG |  |  |  |  |  |
| RP11-254B13.3 | -1.6949196 | 0.000118697 | 0.003080095 | ENST00000414313 | gencode | TTAGACGAGAAAAAGTATCCTTATTGGCCTCACCAACTAATCGAGA |  |  |  |  |  |
| AC016734.2 | -1.6936413 | 0.000165961 | 0.003682421 | ENST00000425312 | gencode | TTTGAATATATCTTGACTAAGCTGCAAGGCGAGGCCCCTTCCAAAA |  |  |  |  |  |
| RP11-692E14.1 | -1.6927667 | 0.005912462 | 0.036315682 | ENST00000510299 | gencode | TCATGCACTAGTCACTGAGAATCTGGTCCCTGGAGAATCAGTTTAT |  |  |  |  |  |
| RP11-314A20.1 | -1.6846387 | 0.000794063 | 0.010130922 | ENST00000466297 | gencode | GTCTCAGGCCAAGGATGACATCGAAGAGTACTTGAAATGCAAGAAA |  |  |  |  |  |
| RPL21P122 | -1.6733584 | 4.72E-05 | 0.001758135 | ENST00000578024 | gencode | ATTCACCAAAATGATGAACACAAAGTGAAAGAGGAGAGGCACCCGA |  |  |  |  |  |
| AC055811.5 | -1.6686804 | 0.004009836 | 0.028553765 | ENST00000423783 | gencode | CAGGAGTAAGATGAGTCAGGCCCCTCCATCATCCACCACAAACGCT |  |  |  |  |  |
| RP11-402G3.4 | -1.6635683 | 0.000284582 | 0.005026687 | ENST00000412010 | gencode | GAAGAAAAAGACCACCCATTTTGTAGAAGGTGGAAATGCTGACAAC |  |  |  |  |  |
| RP1-130G2.1 | -1.6481303 | 0.000304483 | 0.005179586 | ENST00000446953 | gencode | AATTGACTTGCCTACATTGCCTACCCCAAATTTTGGACGTGTGCTT |  |  |  |  |  |
| AC008073.6 | -1.647372 | 0.005087432 | 0.033112075 | ENST00000469867 | gencode | GAGAGGGCGGGGCCTGAGGAGGGCGGAGAGCTGGGGCGGGTCTCAG |  |  |  |  |  |
| RP3-405J24.1 | -1.6391339 | 0.000632262 | 0.008417336 | ENST00000406939 | gencode | ACCGATCTTTAGCCAGAGATCTCACTGGAACCATTAAAGAGATCCT |  |  |  |  |  |
| RPL7AP65 | -1.6270402 | 0.000522227 | 0.007293856 | ENST00000418183 | gencode | GCATTACCAACCTAGAAAAGGCAAAGGCTAAAGAATTTGCCACTAA |  |  |  |  |  |
| AC010761.9 | -1.6189691 | 0.000222576 | 0.004259556 | ENST00000577325 | gencode | TCACTGAAATGGGGGAGGGGCAGGAAAAAGGTTTCTCATTAGGGGA |  |  |  |  |  |
| EPR-1 | -1.6167774 | 0.008892395 | 0.045994109 | uc021uec.1 | UCSC_knowngene | ATAAGAAAGCCATGTTGTTAAACAGTAGAGGAGCCAGGGACTCTGT |  |  |  |  |  |
|  |  |  |  |  |  |  |  |  |  |  |  |
|  |  |  |  |  |  |  |  |  |  |  |  |
| **GeneName** | **FC** | **P-value** | **FDR** | **seqname** | **source** | **Probe sequence** |  |  |  |  |  |
| YBX1P9 | -1.6122585 | 0.003085059 | 0.024221667 | ENST00000441264 | gencode | ATTAAAAAAGAACCCCAGGAAGTACCTTGGCTGTGTAGGAACTGGA |  |  |  |  |  |
| CTA-215D11.4 | -1.5908723 | 0.001235833 | 0.013611949 | ENST00000399343 | gencode | AAGCAGAAAGCCAAGAAAGTGGTGAATCCCCTGTTTAAGAAAAGGC |  |  |  |  |  |
| RP5-817C23.1 | -1.5904821 | 0.008064307 | 0.043427336 | ENST00000426952 | gencode | TGGGGGTGACCAACTGTAGATAATCAAGGATGATGAAAAAGAGGCT |  |  |  |  |  |
| RP4-604A21.1 | -1.5900034 | 0.004237193 | 0.029621652 | ENST00000425821 | gencode | TTTGCGAAAGTTTGTAACTACAATCACCTAATGCCCAAAGCCCGAC |  |  |  |  |  |
| AC007879.5 | -1.5882369 | 0.001630138 | 0.01628978 | ENST00000418850 | gencode | GGGTGAAGTGCTTGATGTCTTTAGTTTATGTTTTCCTAAAACTCCA |  |  |  |  |  |
| RP11-312B8.2 | -1.5877976 | 0.006529139 | 0.038595025 | ENST00000433407 | gencode | TAAAAATTGTACACCACAGATTCAGGCATGGACTCCGTGAGGAAAC |  |  |  |  |  |
| UQCRHP2 | -1.5738024 | 0.000524052 | 0.007293856 | ENST00000415651 | gencode | TAGTGTTTCTACATCAGGATCCCCTAACAACAGTGAGAGAGCAATG |  |  |  |  |  |
| XLOC_007094 | -1.5733806 | 0.006920659 | 0.039983127 | TCONS_00015020 | Cabili et al | GACCAATTAGAGGATTGTAAAATCAGTGTAGTGGGGTGACGAGCAT |  |  |  |  |  |
| RPL24P4 | -1.5715339 | 9.92E-05 | 0.002777045 | ENST00000326586 | gencode | GAATCAGAAACCTGAAGTTAGAAAGACTCGACGAGAACAAGCTATC |  |  |  |  |  |
| RP11-452K12.3 | -1.5642563 | 9.13E-06 | 0.000635301 | ENST00000414870 | gencode | TCACAGATGACATCAACAGTGGTGTTGTGGAATATGCCCAGCTAGT |  |  |  |  |  |
| RP11-64J19.1 | -1.5641892 | 0.000155836 | 0.003618301 | ENST00000585002 | gencode | AACACAAGAACATATGCCAGTTCCTCATAGAGACTGGACTGGCTAA |  |  |  |  |  |
| RPL7P4 | -1.563042 | 0.002157963 | 0.019486618 | ENST00000430239 | gencode | CAACAGGGAGAACCGGATCAACAGACTTATTAGAAGAATGAGCTAA |  |  |  |  |  |
| CASP1P1 | -1.5559859 | 1.92E-07 | 0.000048949 | ENST00000526345 | gencode | TGAAAGGATGACTTTGACAATACGCTTCTACCTCTTCCCAGAACAT |  |  |  |  |  |
| RP11-359M6.3 | -1.5491302 | 0.001218342 | 0.013516529 | ENST00000549457 | gencode | AAGAGCAGCTCCAAGAAGAAAATGTTCCATAAAGAAGCCCAGGAAG |  |  |  |  |  |
| RPL23AP73 | -1.542451 | 9.05E-06 | 0.000635301 | ENST00000492507 | gencode | AGGCAGCAACACACTCATGTTCATTGTGGATGTTAAAGCCAAAAAG |  |  |  |  |  |
| SNHG5 | -1.5386201 | 0.000766577 | 0.009946017 | NR_003038 | RefSeq | CGGGTGGTAGGAACAATGGCGCTGTCTTCAGTGGCACAGTGGAGCA |  |  |  |  |  |
| RPL17P20 | -1.5357737 | 0.00279789 | 0.022664388 | ENST00000461464 | gencode | TTGTTCCTAGACCAGAAAAGGAGGCTACCCAGAAGAAAAAGATATC |  |  |  |  |  |
| RP11-461O14.1 | -1.535008 | 0.000992465 | 0.011688187 | ENST00000469948 | gencode | CTTCTTTGCCTTTGTCAAACTTCAGTGGAAACTTTGTGAAGCTCAA |  |  |  |  |  |
| RP13-93L13.2 | -1.5250465 | 2.08E-05 | 0.001100011 | ENST00000438325 | gencode | TGTACAAATGAGACCCCTCCAAACAGTATGGACTGAAAATGAAAAC |  |  |  |  |  |
| XLOC_002080 | -1.5250087 | 0.003277764 | 0.025356611 | TCONS_00004240 | Cabili et al | TTGTCTCCTTTCCATTACAGCAACTCTTTTGGAGCAAGGGTAATAA |  |  |  |  |  |
| AC013404.1 | -1.502446 | 0.003420702 | 0.026074015 | ENST00000413068 | gencode | ACAGGAGGAAAAGCAAGATTTACAGAAGGATGTTCCTTCAGGAGGA |  |  |  |  |  |
| AC011816.4 | -1.5018691 | 0.000478397 | 0.006845105 | ENST00000494408 | gencode | ACATGCACTTTGCCAAGAAGCACAACAAGAAGGGCCTAAAGAAGAT |  |  |  |  |  |

## Table S4. Top 10 differentially expressed non-redundant Gene Ontology (GO) terms between ETV6-RUNX1-positive and ETV6-RUNX1-negative pediatric B-ALL. For each Gene Ontology term, the genes that were differentially expressed in our samples are shown.

| **UP (ETV6-RUNX1+ vs ETV6-RUNX1-)** | | | |
| --- | --- | --- | --- |
| **Non-redundant GO terms** | **GO group** | **Adjusted p value** | **Genes** |
| regulation of cell proliferation (GO:0042127) | Biological Process | 0.0002 | TFAP2A; FER; ABL2; CD27; TET1; CTCF; GAB2; FGFR2; VEGFA |
| embryonic cranial skeleton morphogenesis (GO:0048701) | Biological Process | 0.0003 | TFAP2A; HOXA1; FGFR2 |
| negative regulation of programmed cell death (GO:0043069) | Biological Process | 0.0016 | TFAP2A; CD27; SERPINB9; ERC1; FGFR2; VEGFA |
| peptidyl-tyrosine modification (GO:0018212) | Biological Process | 0.0036 | FER; ABL2; FGFR2 |
| positive regulation of cellular process (GO:0048522) | Biological Process | 0.0038 | FER; TET1; GAB2; FGFR2; CTGF; VEGFA |
| transmembrane receptor protein tyrosine kinase signaling pathway (GO:0007169) | Biological Process | 0.0068 | FER; ABL2; WASF2; FGFR2; VEGFA |
| positive regulation of stress-activated MAPK cascade (GO:0032874) | Biological Process | 0.0068 | CD27; CTGF; VEGFA |
| peptidyl-tyrosine phosphorylation (GO:0018108) | Biological Process | 0.0068 | FER; ABL2; FGFR2 |
| artery development (GO:0060840) | Biological Process | 0.0071 | HOXA1; VEGFA |
| regulation of mast cell degranulation (GO:0043304) | Biological Process | 0.0071 | FER; GAB2 |
|  |  |  |  |
|  |  |  |  |
|  |  |  |  |
|  |  |  |  |
|  | | | |
| **DOWN (ETV6-RUNX1+ vs ETV6-RUNX1-)** | | | |
| **Non-redundant GO terms** | **GO group** | **Adjusted p value** | **Genes** |
| regulation of apoptotic process (GO:0042981) | Biological Process | 3.3·10^-12^ | MAP2K3; MAP2K4; MOAP1; FLT1; SEMA4D; GADD45A; STRADA; ACTN1; PAWR; TNFRSF10A; CFLAR; STK3; NFKBIA; FLCN; NLRP12; TP53I3; ERBB4; KDR; BNIP1; PIM1; ABL2; CASP2; BIRC5 |
| protein phosphorylation (GO:0006468) | Biological Process | 1.9·10^-6^ | FLT1; PRKCE; MTOR; STK3; TGFBR3; BCR; CREB1; ERBB4; KDR; PIM1; ABL2; BIRC5; TLR4 |
| activation of protein kinase activity (GO:0032147) | Biological Process | 1.9·10^-6^ | MAP2K3; MAP2K4; TRAF7; GADD45A; STRADA; RAPGEF1; TNFRSF10A; TLR4; MTOR; STK3 |
| Rab protein signal transduction (GO:0032482) | Biological Process | 3.1·10^-5^ | RAB2A; RAB10; RAB26; RAB8A; RAB40AL; RAB7A |
| protein kinase activity (GO:0004672) | Molecular Function | 3.9·10^-5^ | MAP2K3; BCR; MAP2K4; FLT1; ERBB4; STRADA; PRKCE; KDR; PIM1; ABL2; MTOR; STK3 |
| cellular response to oxygen-containing compound (GO:1901701) | Biological Process | 6.7·10^-5^ | RAP1B; EGR1; IGFBP5; PXN; RAPGEF1; CARD17; ABL2; TLR4; MTOR |
| cellular response to mechanical stimulus (GO:0071260) | Biological Process | 9.0·10^-5^ | MAP2K4; GADD45A; CASP2; TNFRSF10A; TLR4 |
| GDP binding (GO:0019003) | Molecular Function | 9.3·10^-5^ | RAB2A; RAP1B; RAB10; RAB8A; RAB7A |
| regulation of cell cycle (GO:0051726) | Biological Process | 9.5·10^-5^ | MAP2K3; MAP2K4; GADD45A; STRADA; ID4; PIM1; BIRC5; STK3 |
| Ras protein signal transduction (GO:0007265) | Biological Process | 1.0·10^-4^ | RAB2A; RAP1B; RAB10; RAB26; RAPGEF1; RAB8A; RAB40AL; RAB7A |

## Table S5. Pairs of long non-coding RNAs (lncRNAs) and associated messenger RNAs (mRNAs) differentially expressed between ETV6-RUNX1-positive and ETV6-RUNX1-negative pediatric B-ALL.

| **Differentially expressed lncRNAs** | | | | **Distance** | **Associated differentially expressed mRNAs** | | | |
| --- | --- | --- | --- | --- | --- | --- | --- | --- |
| **Probe ID** | **Gene symbol** | **Adj. p** | **FC** |  | **Probe ID** | **Gene symbol** | **Adj. p** | **FC** |
| ASPWP0008581 | TCL6 | 7.76·10^-4^ | 3.80 | 12964 | ASPWP0007741 | TCL1B | 1.10·10^-4^ | 20.81 |
| ASPWP0008581 | TCL6 | 7.76·10^-4^ | 3.80 | 12964 | ASPWP0010107 | TCL1B | 6.62·10^-3^ | 4.42 |
| ASPWP0005785 | RP11-345I18.1 | 1.76·10^-3^ | 2.62 | 0 | ASPWP0003168 | ABL2 | 7.22·10^-2^ | 1.51 |
| ASPWP0005787 | EIF4A1P11 | 1.01·10^-4^ | -2.03 | 0 | ASPWP0003168 | ABL2 | 7.22·10^-2^ | 1.51 |
| ASPWP0002552 | BC045725 | 2.94·10^-2^ | 1.93 | 204 | ASPWP0010032 | INTS6 | 3.64·10^-2^ | 2.43 |
| ASPWP0234632 | D28359 | 6.26·10^-4^ | -1.91 | 5820 | ASPWP0010032 | INTS6 | 3.64·10^-2^ | 2.43 |
| ASPWP0129921 | AC016700.6 | 1.76·10^-3^ | -1.87 | 5207 | ASPWP0007257 | TIA1 | 3.51·10^-2^ | 1.94 |
| ASPWP0129921 | AC016700.6 | 1.76·10^-3^ | -1.87 | 5207 | ASPWP0010541 | TIA1 | 7.80·10^-2^ | 2.14 |
| ASPWP0101740 | RP11-226F19.1 | 5.80·10^-3^ | -1.82 | 43783 | ASPWP0009263 | ACTN1 | 1.07·10^-4^ | -2.37 |
| ASPWP0005745 | RP11-69I8.3 | 3.57·10^-2^ | 1.79 | 0 | ASPWP0011091 | CTGF | 2.39·10^-2^ | 3.11 |
| ASPWP0132347 | AC007563.4 | 1.25·10^-3^ | -1.76 | 90224 | ASPWP0010578 | IGFBP5 | 2.58·10^-2^ | 1.66 |
| ASPWP0231913 | EIF4A1P7 | 1.09·10^-4^ | -1.73 | 103704 | ASPWP0002452 | FLT1 | 2.64·10^-3^ | 1.64 |
| ASPWP0134505 | RP1-159A19.4 | 3.66·10^-2^ | 1.67 | 35637 | ASPWP0001625 | WASF2 | 2.92·10^-4^ | 1.92 |
| ASPWP0089678 | RP5-817C23.1 | 4.34·10^-2^ | -1.59 | 24161 | ASPWP0009349 | GADD45A | 2.74·10^-3^ | 1.88 |
| ASPWP0089678 | RP5-817C23.1 | 4.34·10^-2^ | -1.59 | 24161 | ASPWP0004769 | GADD45A | 2.74·10^-3^ | 1.83 |
| ASPWP0171783 | AC002454.1 | 7.25·10^-2^ | -1.58 | 18281 | ASPWP0005846 | CDK6 | 5.01·10^-2^ | 1.63 |
| ASPWP0007620 | CD27-AS1 | 7.62·10^-4^ | 1.57 | 0 | ASPWP0002233 | CD27 | 1.17·10^-5^ | 6.26 |
| ASPWP0194434 | CASP1P1 | 4.89·10^-5^ | -1.56 | 21229 | ASPWP0011542 | CARD17 | 4.67·10^-3^ | -1.67 |
| ASPWP0154018 | RPL17P20 | 2.27·10^-2^ | -1.54 | 28353 | ASPWP0005046 | HOPX | 2.80·10^-2^ | -1.61 |

The cutoffs were fold change (absolute value) > 1.5 and FDR < 0.1 for both the lncRNA and the associated protein-coding gene. The table is sorted according to the fold change of the lncRNAs. “Adj. p”: false discovery rate (FDR) adjusted p value. “FC”: fold change. “Distance”: genomic distance between the lncRNA and the associated protein-coding gene.

## Table S6. Number of pediatric B-ALL patients in the internal and external cohorts used in our analyses.

| **Number of samples** | **Our cohort** | **Fernando et al (2015)** | **Ghazavi et al (2016)** | **Lee et al (2015)** |
| --- | --- | --- | --- | --- |
| Healthy samples | 4 | 0 | 0 | 0 |
| Normal karyotype | 7 | 0 | 17 | 0 |
| Hyperdiploid | 8 | 0 | 15 | 31 |
| MLL translocations | 0 | 15 | 0 | 0 |
| TCF3-PBX1 | 0 | 15 | 7 | 1* |
| ETV6-RUNX1 | 24 | 14 | 25 | 24 |
| Other / ND | 3 | 0 | 0 | 25 |

ND: not determined. *Sample was removed from the analyses because no statistical test could be performed for N = 1.

## Table S7. Kruskal-Wallis and Wilcoxon rank-sum p values for the differential expression of TCL6 and TCL1B in all possible comparisons between pediatric B-ALL subtypes in internal and external cohorts. All p values were adjusted for multiple comparisons using the Benjamini-Hochberg method. Significant p values are marked in green.

| **TCL6** | | | | | | | |
| --- | --- | --- | --- | --- | --- | --- | --- |
| **Study** | **Kruskal-Wallis** | **ETV6-RUNX1 vs MLL** | **ETV6-RUNX1 vs TCF3-PBX1** | **ETV6-RUNX1 vs Normal karyotype** | **ETV6-RUNX1 vs Hyperdiploid** | **ETV6-RUNX1 vs Other** | **ETV6-RUNX1 vs healthy** |
| Fernando et al (2015) | 1.31·10^-4^ | 4.01·10^-3^ | 8.80·10^-1^ | NA | NA | NA | NA |
| Ghazavi et al (2016) | 5.31·10^-3^ | NA | 7.09·10^-1^ | 7.88·10^-2^ | 1.14·10^-2^ | NA | NA |
| Lee et al (2015) | 8.30·10^-5^ | NA | NA | NA | 3.68·10^-5^ | 1.74·10^-3^ | NA |
| Our data | 1.31·10^-4^ | NA | NA | 7.70·10^-3^ | 1.17·10^-3^ | NA | 9.77·10^-4^ |
| **TCL1B** | | | | | | | |
| **Study** | **Kruskal-Wallis** | **ETV6-RUNX1 vs MLL** | **ETV6-RUNX1 vs TCF3-PBX1** | **ETV6-RUNX1 vs Normal karyotype** | **ETV6-RUNX1 vs Hyperdiploid** | **ETV6-RUNX1 vs Other** | **ETV6-RUNX1 vs healthy** |
| Fernando et al (2015) | 5.75·10^-5^ | 4.21·10^-5^ | 4.46·10^-3^ | NA | NA | NA | NA |
| Ghazavi et al (2016) | 1.31·10^-4^ | NA | 8.04·10^-2^ | 7.70·10^-3^ | 2.98·10^-4^ | NA | NA |
| Lee et al (2015) | 1.31·10^-4^ | NA | NA | NA | 2.98·10^-4^ | 1.74·10^-3^ | NA |
| Our data | 8.63·10^-5^ | NA | NA | 7.38·10^-3^ | 1.17·10^-3^ | NA | 9.77·10^-4^ |

# Supplementary figure legends.

**Figure S1. Unsupervised hierarchical clustering of pediatric B-ALL samples and healthy bone marrow based on lncRNA expression profiles**. Samples and genes were clustered based on the Spearman correlation coefficient. On top of the heatmap, samples from healthy donors are labeled in green, ETV6-RUNX1-negative B-ALL samples are labeled in blue and ETV6-RUNX1-positive B-ALL samples are labeled in red.

**Figure S2. Gene Ontology (GO) analysis of the mRNAs associated with the differentially expressed lncRNAs in ETV6-RUNX1-positive *vs.* ETV6-RUNX1-negative pediatric B-ALL**. The top 10 significantly enriched GO terms among the mRNAs associated with the upregulated (UP) or downregulated (DOWN) lncRNAs are shown. Only non-redundant GO terms are included as described in Supplementary Methods.

**Figure S3**. **Normalized microarray expression values of the 16 lncRNA/mRNA pairs that were differentially expressed between ETV6-RUNX1-positive and ETV6-RUNX1-negative pediatric B-ALL**. For each lncRNA/mRNA pair, the distribution of lncRNA expression values is shown on the left and the distribution of the associated mRNA expression values is shown on the right. Samples are classified as healthy (green), ETV6-RUNX1-negative pediatric B-ALL (blue) and ETV6-RUNX1-positive pediatric B-ALL (red). FDR-adjusted Kruskal-Wallis p-values are shown on top of each of the comparisons. Significant p-values (p < 0.05) are highlighted in bold.

**Figure S4**. **Quantitative polymerase chain reaction validation of the differential expression of TCL6 and TCL1B between ETV6-RUNX1-positive and ETV6-RUNX1-negative B-ALL samples from our microarray study.** Three samples per group were used for the validation.
