# Supplementary figures and images for: Expression of the long non-coding RNA TCL6 is associated with clinical outcome in pediatric B-cell acute lymphoblastic leukemia
Source: Blood Cancer J. 2019 Nov 25;9(12):93. doi: 10.1038/s41408-019-0258-9 (PMC6877621; doi:10.1038/s41408-019-0258-9)

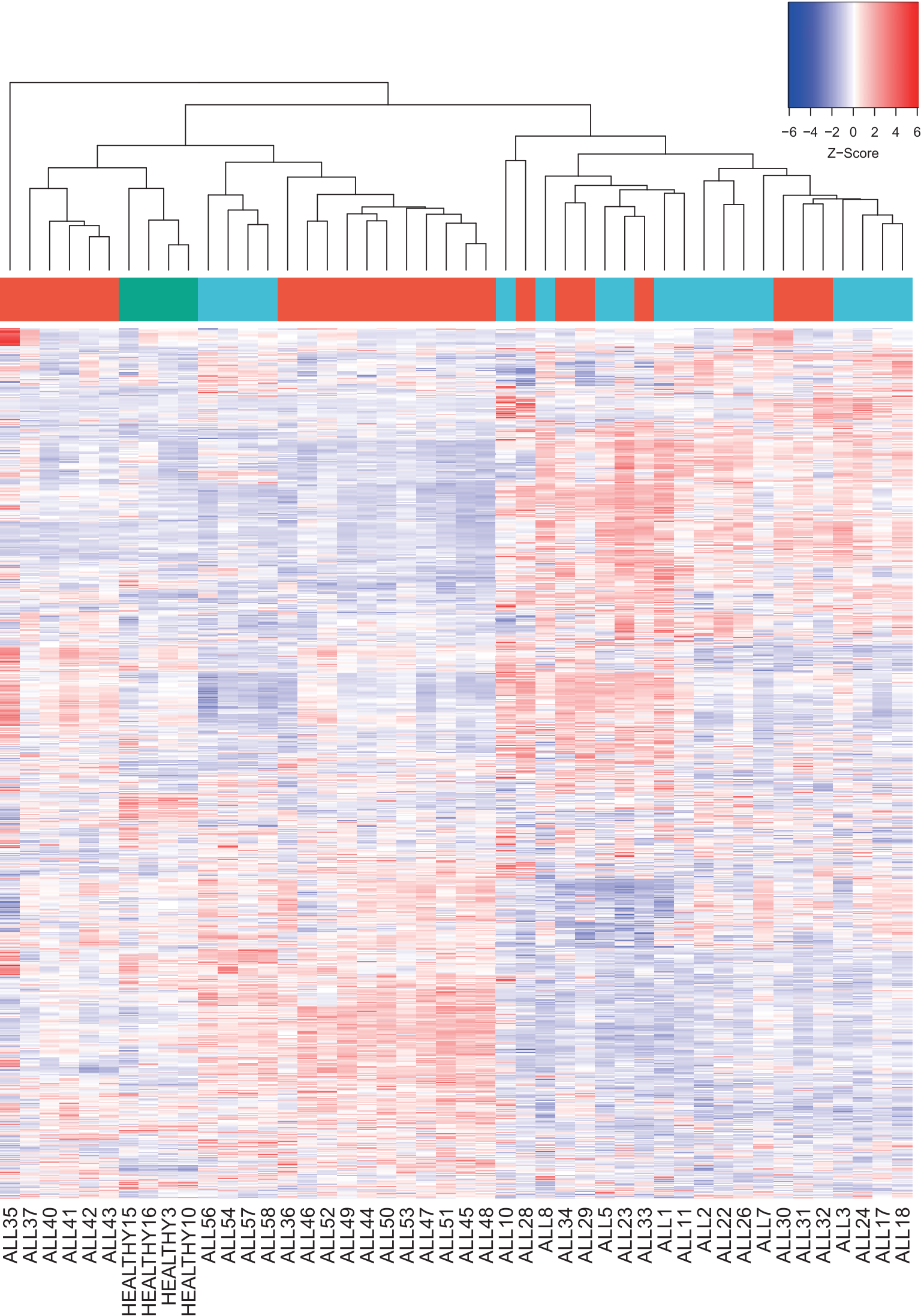

Supplement: Supplementary file 2 — Figure S1 [file 41408_2019_258_MOESM2_ESM.pdf]

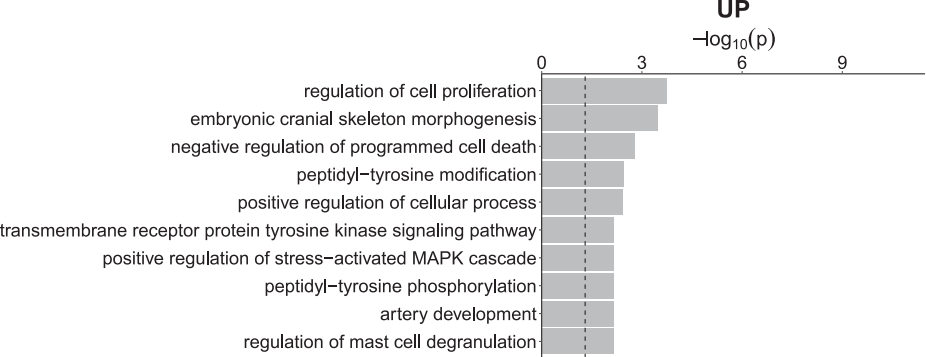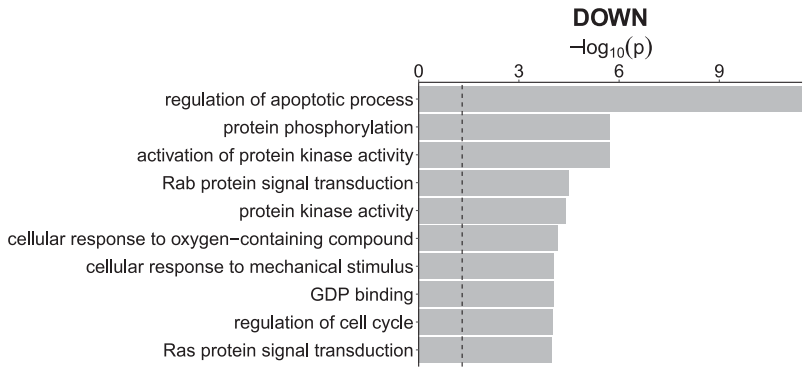

Supplement: Supplementary file 3 — Figure S2 [file 41408_2019_258_MOESM3_ESM.pdf]

**Sample type** ■ Healthy ■ ETV6-RUNX1- ■ ETV6-RUNX1+

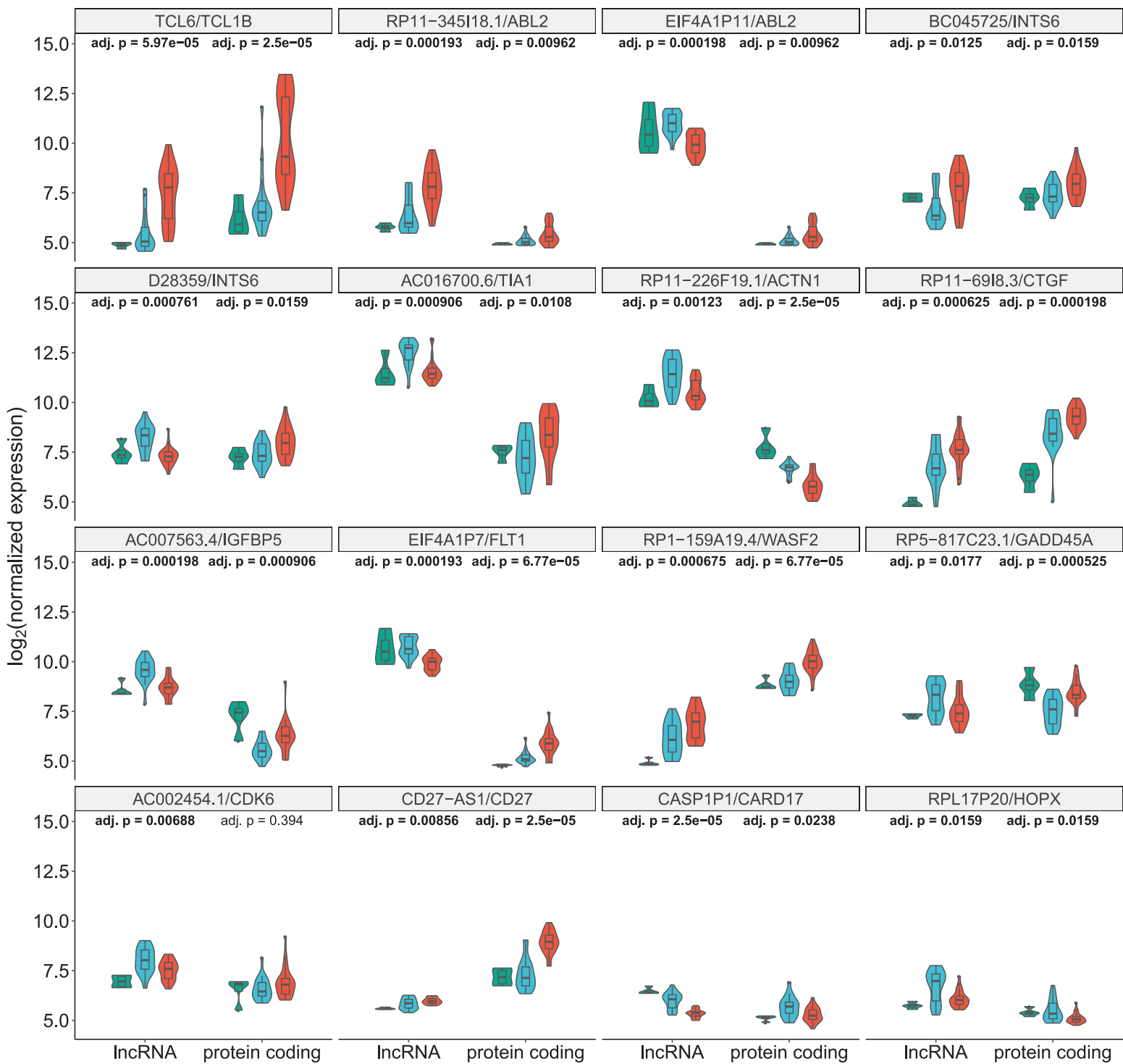

Supplement: Supplementary file 4 — Figure S3 [file 41408_2019_258_MOESM4_ESM.pdf]

log2(expression relative to GAPDH)

TCL6

T-test,  $p = 0.0015$

2

0

-2

TCL1B

T-test,  $p = 0.01$

2

0

-2

ETV6-RUNX1- ETV6-RUNX1+  
Patient group

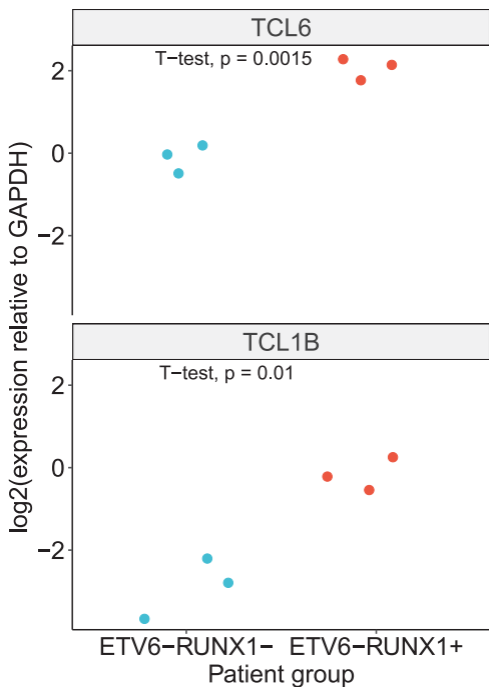

Supplement: Supplementary file 5 — Figure S4 [file 41408_2019_258_MOESM5_ESM.pdf]
